# How to Manage a Neonate Born from a SARS-CoV-2-Positive Mother: A Narrative Review

**DOI:** 10.3390/pathogens13110977

**Published:** 2024-11-08

**Authors:** Serena Salomè, Ida D’Acunzo, Federica Fanelli, Simona Perniciaro, Letizia Capasso, Francesco Raimondi, Chryssoula Tzialla, on behalf of the Study Group of Neonatal Infectious Diseases

**Affiliations:** 1 Division of Neonatology, Department of Translational Medical Sciences, University of Naples Federico II, Via Pansini 5, 80131 Naples, Italy; ida.dacunzo@gmail.com (I.D.); ricafede91@gmail.com (F.F.); letizia.capasso@unina.it (L.C.); raimondi@unina.it (F.R.); 2 Neonatal Intensive Care Unit, “Filippo del Ponte” Hospital, ASST Settelaghi, 21100 Varese, Italy; simona.perniciaro@asst-settelaghi.it; 3 Neonatal and Pediatric Unit, Polo Ospedaliero Oltrepò, ASST Pavia, 27058 Voghera, Italy; chryssoula.tzialla@unipv.it

**Keywords:** SARS-CoV2, COVID-19, neonatal outcome, MIS-N

## Abstract

From 2020 to today, considerable knowledge on SARS-CoV-2 has been collected, even on pregnant women and their fetuses and newborns, and clinical guidelines have been written and implemented worldwide. Vaccination has considerably improved outcomes, but hesitancy amongst pregnant patients and the emergence of variants remain challenging, and SARS-CoV-2 positivity during pregnancy continues to be associated with an increased risk of maternal complications, premature delivery, and higher neonatal mortality and morbidity. A body of data now exists on the effect of SARS-CoV-2 during pregnancy on early neonatal outcomes, medical education in obstetrics and pediatrics, and longer-term developmental outcomes. This review aimed to present important findings on clinical outcomes and health recommendations for neonate born from a SARS-CoV-2-positive mother in order to summarize effective preventive healthcare guidelines.

## 1. Introduction

Severe Acute Respiratory Syndrome Coronavirus 2 (SARS-CoV-2) was first isolated in Wuhan, China, at the end of 2019. The virus’s high contagiousness caused its rapid spread, affecting 199 countries worldwide and, therefore, on 11 March 2020, the World Health Organization (WHO) declared the beginning of the pandemic [1]. The spread of the infection was extremely wide, and 775,917,102 cases and 7,058,381 deaths have been registered around the world up to 31 July 2024 [2].

At the beginning of the pandemic, one of the most challenging questions was whether a vertical/perinatal transmission of the infection was possible and the potential adverse effects on fetuses and neonates as well on pediatric outcomes, both early and longer-term. 

In the first months of the pandemic, contrasting guidelines were recommended, from the very strict regime in China, consisting of encouraging cesarean section, avoiding contact between mother and child, and withholding both breastfeeding and expressed breast milk [3], to the middle way of the American Academy of Pediatrics (AAP) and the USA Centers for Disease Control and Prevention (CDC) that advocated routine delivery, separation between mother and child, and encouraged the use of expressed breast milk fed by alternate caregiver [4,5], ending with the WHO [6] and European organizations (such as the Union of European Neonatal and Perinatal Societies (UENPS)) [7], which recommended keeping mothers and infants together and suggested direct breastfeeding after breast hygiene. Afterwards, more uniform recommendations were adopted across the world because of the increasing knowledge on the topic and the outcomes of the different approaches. Both vertical and horizontal transmissions emerged to be rarer than supposed, and it was demonstrated that antibodies are transferred from mothers to infants through the placenta and breast milk. Infected infants appeared to be mildly symptomatic, even though recent data showed multisystem inflammatory syndrome and delayed developmental outcomes in neonates exposed to SARS-CoV-2 in utero. 

The vaccination of pregnant women changed dramatically the prognosis of this group of population and of their children subsequently, despite a persisting percentage of vaccine hesitancy and the emergence of viral variants. 

Therefore, this review aimed to present important findings on clinical outcomes and health recommendations for neonates born from SARS-CoV-2-positive mothers in order to summarize effective preventive healthcare guidelines and improve their outcome and reduce irrational attitudes based on fears as in the first pandemic months, with extensive use of cesarean section, formula feeding, and separation from mothers.

## 2. Recommendations for Maternal Vaccination

It is well-documented that pregnant women are at an increased risk of experiencing severe symptoms and mortality compared to non-pregnant adults, especially those affected by gestational diabetes mellitus (GDM) and pre-eclampsia [8]. Therefore, the primary goal of vaccination is to protect pregnant women from infection, particularly the severe forms, and to protect their children as a secondary outcome. In fact, the maternal administration of mRNA vaccines stimulates both humoral and cell-mediated immune responses in the woman, providing protection for her, and facilitates the transfer of IgG antibodies through the umbilical cord to the fetus, potentially avoiding the risk of infection in the newborn in the first months of life [9,10,11]. To a lesser extent, maternal vaccination during pregnancy or breastfeeding also leads to the transfer of IgG and IgA antibodies to newborns fed with their own mother’s milk [12,13].

The recommendations regarding SARS-CoV-2 vaccination during pregnancy and lactation have been repeatedly updated. After initial caution due to limited scientific evidence, it is now widely recommended for mothers to get vaccinated during both of these periods [14,15]. Vaccination improves pregnancy outcomes both for mothers and newborns, such as lower risks of postpartum hemorrhage and chorioamnionitis [16], severe neonatal morbidity, intrauterine fetal and neonatal death, and neonatal intensive care unit admission with no increase in neonatal readmission or hospital admission up to age of 6 months [17] and preterm birth, small for gestational age status, or low Apgar score [16]. Furthermore, first-trimester mRNA COVID-19 vaccine exposure was not associated with an increased risk for selected major structural birth defects [18,19]. In addition, boosters appear to confer additional protection for pregnant women, reducing stillbirth without increasing the rates of preterm birth or very-low birthweight neonates compared to vaccinated but unboosted women [20].

Therefore, vaccination during pregnancy is safe and effective and may also protect infants, particularly those aged <3 months, after delivery against COVID-19-related hospitalizations [10]. 

If the mother has not been vaccinated previously because the opportunity to receive a COVID-19 vaccine was missed or she declined it during pregnancy, vaccination should be encouraged after delivery, even during breastfeeding (primary cycle and booster dose) without the need to withhold it [21]. In fact, specific studies in this population have not revealed significant safety concerns, demonstrating no harm during lactation but rather the presence of specific antibodies in the breast milk of vaccinated women [14]. There may be an occasional brief reduction in milk production for about 48–72 h after vaccination. The mRNA is rapidly degraded without entering the cell nucleus and, although rare cases of detection of SARS-CoV-2 RNA in breast milk have been reported, the virus has not been cultured from these samples, suggesting a low risk of transmission to the breastfed baby [13]. Regarding efficacy, the available data indicate that specific IgA antibodies are present in breast milk in greater amounts after the administration of mRNA vaccines compared to viral vector vaccines. Current studies suggest that the antibody protection provided to the newborn by the mother is greater if the mother is vaccinated during pregnancy than during breastfeeding.

Despite the existence of studies on safety and improved outcomes, a relevant number of pregnant women remain unvaccinated, often due to vaccine hesitancy related to religious concerns, socioeconomic factors, inadequate information, and lack of trust towards medical professionals [22]. Furthermore, and regrettably, resistance to vaccination has been demonstrated also among healthcare workers, including those in maternity settings [23]. 

## 3. Mother-to-Child Transmission of SARS-CoV-2

In February 2021, the WHO drafted a specific document [24] where mother-to-child transmission (MTCT) was categorized based on three elements: (1) the timing of the documented maternal infection; (2) tests to evaluate the likelihood of early in utero or intrapartum exposure; and (3) tests to evaluate the later exposure/persistence of the virus or virus-specific immune response in the fetus/neonate. As summarized in Table 1, MTCT can be secondary to:

1. In utero transmission.
2. *Intrapartum* transmission.
3. Early *post-natal* transmission (>48 h—28 days).

Moreover, according to the WHO, MTCT is classified as: (1) confirmed; (2) possible (evidence is suggestive but not confirmatory for infection); (3) unlikely (little support for diagnosis but infection cannot be completely ruled out); and (4) indeterminate (when the tests required to establish the class have not been performed).

Suspected cases of COVID-19 are defined by the most current definitions as well as the local standards and protocols [25].

The vertical transmission of SARS-CoV-2 can occur through two different mechanisms: entering the placenta through transmembrane serine protease2 by binding to ACE2 or crossing it through infected macrophages and monocytes [26].

Regardless of the type of transmission (vertical or horizontal), positivity rates in offspring using RT-PCR varied between regions, ranging from 0.1% in North America to 8.5% in Latin America and the Caribbean, with an overall prevalence of 2.7% [27,28]. The types and timing of the tests used to diagnose SARS-CoV-2 infection in babies varied between studies. The clinical outcomes of babies born to mothers with SARS-CoV-2 infection were inconsistently reported. 

The vertical transmission of the virus is possible, though rare, with recent studies reporting that the risk is 2.1% [29] (between 0.8 and 3% [21]), more frequently associated with severe maternal clinical features and maternal infection with the Delta virus. Horizontal transmission is also relatively rare and described in 0.1 % of neonates [30].

Regarding the intrapartum transmission of the virus, its presence in vaginal fluids is rare, but SARS-CoV-2 RNA has been identified in the stools of several infected individuals (43% of examined cases) [31], suggesting the possibility of pathogen spread by contiguity during vaginal delivery [32,33].

The possible contamination of the surrounding environment by maternal droplets or aerosols, as well as maternal fecal material, makes it difficult to accurately distinguish between actual vertical transmission and possible early horizontal transmission [33]. SARS-CoV-2 RT-PCR positivity has been demonstrated in the cord blood (3.6%), placenta (7.7%), and in fecal and rectal samples of pregnant women (9.7%) as well as through serological data (IgM-positive in 3.7% of cases) [34].

Regarding postnatal transmission through breast milk, viral RNA has been detected in the milk using real-time PCR techniques. However, this appears to be a rare occurrence, and no correlation has been identified between the presence of viral RNA and infection in the neonate [35].

## 4. Diagnostic Tests in Neonatal Age

- Virological tests: The positivity of PCR for SARS-CoV-2 confirms the presence of the virus but not whether it is replicating. A single positive nasopharyngeal swab may indicate different situations, such as active viral replication and the presence of viral fragments acquired during passage through the birth canal or in the immediate postpartum or transient contamination [36]. The interpretation of a single positive nasopharyngeal swab is complex, and the persistence of a positive test or a positive test from normally sterile samples (i.e., neonatal blood, lower respiratory tract samples, and cerebrospinal fluid) can be decisive. The sensibility of nasopharyngeal PCR is limited and varies from 53% to 86%. If the symptoms are suggestive of infection during a period of increasing prevalence of COVID-19 and if there is known contact with a patient affected by COVID-19, in the case of negativity, the repetition of the nasopharyngeal swab is indicated [37].
- Serologic tests: Neonatal IgG reflects the transplacental passage of maternal immunoglobulins. Maternal IgM and IgA usually do not cross the placenta, but the sensibility and specificity of the IgM test are lower than the molecular test, and false positive and negative IgM tests have been reported. Nevertheless, the presence of IgM in the newborn in the first 7 days of life may reflect fetal infection, while after 7 days, it may support the diagnosis of intrapartum or post-natal infection [38].

In conclusion, the diagnosis in newborns is based on a nasopharyngeal swab for SARS-CoV-2, while serological tests (IgM, IgG, or total antibody measurement) are not routinely recommended. Moreover, asymptomatic newborns with SARS-CoV-2 infection do not require additional laboratory tests and instrumental examinations, which are instead suggest for symptomatic newborns based on their clinical condition to evaluate potential complications and exclude alternative diagnoses.

## 5. Pregnancy Outcomes

An increasing number of pregnant women positive for SARS-CoV-2 have been reported globally, with a trend similar to that of the general population.

After initial impressions that SARS-CoV-2 infection had a more benign course in pregnant women compared to other populations, the progressively collected data demonstrated that SARS-CoV-2 infection in this group is associated with an increased risk of severe disease and obstetric complications, such as pre-eclampsia/eclampsia, gestational diabetes, and thrombosis [39]. Infected pregnant women have double the risk of being admitted to intensive care, needing mechanical ventilation, and extracorporeal oxygenation; mortality is also higher compared to that of non-infected pregnant women [40]. Factors such as advanced maternal age, obesity, pre-existing comorbidities, chronic hypertension, diabetes, and pre-eclampsia are associated with severe disease [27]. The risk of miscarriage, intrauterine growth restriction, and preterm birth are double than in non-infected pregnant women, making the infection risky not only for the pregnant woman but also for the unborn child.

Data collected in the United Kingdom through the PAN-COVID study (Pregnancy and Neonatal Outcomes for Women with COVID) and in the USA through the Perinatal COVID-19 Registry (American Academy of Pediatrics (AAP) Section on Neonatal-Perinatal Medicine [SONPM] National Perinatal COVID-19 Registry) showed: maternal mortality, 0.5%; early neonatal mortality, 0.2%; miscarriage rate, 0.5%; prematurity (less than 37 weeks’ gestation), 16%, with extreme prematurity (less than 27 weeks’ gestation), 0.5%; neonatal infection, approximately 2%; and low birth weight (SGA), 9.7% [36].

In general, fetal and neonatal outcomes (in terms of increased risk of miscarriage, intrauterine death, and prematurity) [41] may depend more on the severity of maternal infection and concurrent obstetric pathologies than on SARS-CoV-2 infection transmitted from the mother to the fetus, similar to past SARS-CoV-1 (Severe Acute Respiratory Syndrome) and MERS-CoV (Middle East Respiratory Syndrome Coronavirus) epidemics, also considering that respiratory viruses are not easily transmitted in utero [42].

Several fetal conditions may have been attributed to COVID-19 in pregnancy, such as myocarditis, tachycardia, the calcification of fetal bowel and bladder, periventricular leukomalacia, cerebral venous thrombosis, cerebral ischemic lesions, neonatal necrotizing enterocolitis, and infantile immune thrombocytopenia [26]. 

SARS-CoV-2 positivity in pregnant women is not an indication for cesarean delivery [21]. 

## 6. Delivery Room Management of Neonates Born to Mothers with SARS-CoV-2 Infection at the Time of Delivery

All healthcare providers should be protected from acquiring the infection during the delivery, whether spontaneous or cesarean, of patients with suspected or confirmed COVID-19 infection, applying “transmission-based precautions” by using appropriate personal protective equipment (PPE), including gown and gloves, an N95 respirator, and eye protection (goggles or face shield), or an air-purifying respirator that provides eye protection, at least until the neonate’s negativity is confirmed [43,44].

### 6.1. Resuscitation

Neonatal resuscitation should follow the standard guidelines of the Neonatal Resuscitation Program (NRP) [45,46], providing positive pressure ventilation (through a bag-mask or a T-piece/mask) with healthcare workers using appropriate PPE to protect themselves. In fact, they are particularly at risk during resuscitation maneuvers because SARS-CoV-2 is a highly transmissible virus and cardiopulmonary resuscitation (CPR) is considered an aerosol-generating procedure, with viral particles, which can remain suspended in the air, being subsequently inhaled during chest compressions, positive pressure ventilation, and the placement of a ventilatory support, with a half-life of about an hour [47].

Resuscitation also requires numerous healthcare providers working closely together and with the patient, and stress can result in the failure to apply all infection control strategies. Even if cases of neonates infected at birth are rare, those assisting such neonates must wear appropriate personal protective equipment. Additionally, the mother is a potential source of aerosols for the neonatal team [47]. It would be appropriate for the newborn’s care at birth to take place in the delivery room where the mother is accommodated or in a dedicated neonatal area, both equipped with complete facilities for proper primary resuscitation. 

The subsequent transport of the newborn from the delivery room to the rooming-in area or to the isolation rooms set up for the child of a positive mother should preferably be conducted using a closed cradle (see Table 2).

### 6.2. Cord Clamping

The benefits of delayed clamping are known: an increase in immunobiological factors and neonatal hemoglobin as a result of physiological placental transfusion and improved neurodevelopment [48]. However, during the Chinese phase of the COVID-19 pandemic, immediate cord clamping was implemented to reduce the risk of the intrapartum transplacental transmission of the infection [49]. Current knowledge suggests that the vertical transmission of SARS-CoV-2 was similarly low (<1.5%) following both delayed and early cord clamping [50]. Thus, delayed cord clamping should be practiced for all stable neonates, even if the mother is infected [21].

### 6.3. Skin-to-Skin Contact

Skin-to-skin contact (SSC) should be practiced for all stable neonates, even if born to positive mothers, provided the mothers are asymptomatic or have mild symptoms. SSC by a COVID-19-positive mother is encouraged [51], using a face mask while holding her infant in order to reduce the risk of postnatal transmission, in addition to scrupulously following respiratory prevention measures and proper hand washing [44]. The positive effects of SSC are well known, particularly in initiating breastfeeding and bonding [52], and initial doubts about its safety have now been dispelled.

There is no reason to consider SSC a dangerous practice for the transmission of infection from a COVID-19-positive mother to her neonate. Both the amniotic fluid [34] and vaginal secretions [53] of women are almost always negative for SARS-CoV-2. The oro-fecal transmission route [54] is hypothesized, while transmission through the vaporization of biological fluids [55] is not well documented. It may still be good practice to clean the mother’s abdomen and the neonate of secretions or blood before starting SSC.

It seems contradictory to allow intimate contact between mother and child during feeding but deny it during SSC, even though, in both cases, proximity increases the risk of respiratory transmission. However, this risk is mitigated if mothers have appropriate protective measures for the neonate. At the time of delivery, however, the mother may have difficulty in correctly following prevention measures, such as properly wearing a mask.

## 7. Breastfeeding

Mothers with suspected or confirmed COVID-19 should be encouraged to initiate or continue to breastfeed, if their clinical condition allows it and respecting their desire to feed the baby directly at the breast or through the manual or mechanical expression of breast milk [6]. 

In the case of a premature newborn or other neonatal or maternal diseases, the mother should be encouraged and supported to practice milk expression. Procedures to reduce the risk of infectious transmission, such as wearing a mask especially during breastfeeding, hand hygiene, and the regular cleaning and disinfection of surfaces, should be adopted [4]. 

The mothers of infants in the NICU should express breast milk when their infection status prohibits their presence in the NICU. Healthcare workers should use transmission-based precautions when caring for infants who are well in the same room as a person with COVID-19 [21]. 

The pasteurization of milk from a mother with SARS-CoV-2 infection is not indicated, as breast milk is not considered a vehicle for the virus. Furthermore, pasteurization would reduce the biological and immunological value of human milk [56].

The compatibility of breastfeeding with specific anti-COVID-19 medications administered to the mother should be evaluated on a case-by-case basis.

Several published studies have detected SARS-CoV-2 nucleic acid in breast milk but not the viable infectious virus. One study demonstrated that pasteurization methods (such as those used to prepare donor milk) inactivate SARS-CoV-2. Moreover, specific IgA and IgG antibodies have been detected in breast milk after both maternal infection and maternal vaccination against SARS-CoV-2. 

Therefore, direct breastfeeding is encouraged with these recommendations [44]:

- Infected mothers should perform hand hygiene before breastfeeding and wear a mask during it.
- If an infected mother decides not to breastfeed her newborn, she may express milk so that an uninfected caregiver can administer it to the infant.
- Expressing milk is suggested to mothers of infants in the NICU whenever their infection status prohibits their presence in the NICU.

## 8. SARS-CoV-2 Infection in the Neonatal Period

Respiratory infections caused by common coronaviruses in the neonatal period and, more generally, in the first year of life were already known even before the current SARS-CoV-2 pandemic [57,58].

It is important to distinguish between cases of positivity within the first 48 h (vertical transmission) and those in later periods (horizontal transmission), even if within the first month of life. These data could change with the emergence of new variants with different infectivity. Positivity that occurs in the first 2–3 days of life is likely attributable to ineffective control measures for transmission from the mother to the neonate, rather than vertical transmission. The most significant portion of infected neonates is represented by those who become positive at home during the first month of life due to contact with a SARS-CoV-2-positive family member rather than via the transplacental route. These neonates may require hospitalization if symptomatic, but the disease is rarely severe [21].

Despite the possibility that SARS-CoV-2-positive neonates may develop respiratory failure and require intensive care, generally, the clinical course of infection at this age is favorable, although prematurity may be a factor that determines a greater clinical severity [21]. In fact, most infected neonates were asymptomatic or developed a mild illness, with tachypnea and fever as the most common signs, without the need for respiratory support. Only <8% had severe infection, and they were more likely to receive respiratory support [30].

Although neonatal morbidity is low both from vertical and horizontal transmissions, a newly recognized clinical entity called multisystem inflammatory syndrome emerged, associated with maternal or neonatal SARS-CoV-2 infection [30].

### 8.1. Multisystem Inflammatory Syndrome in Neonates (MIS-N)

**Figure 1:**
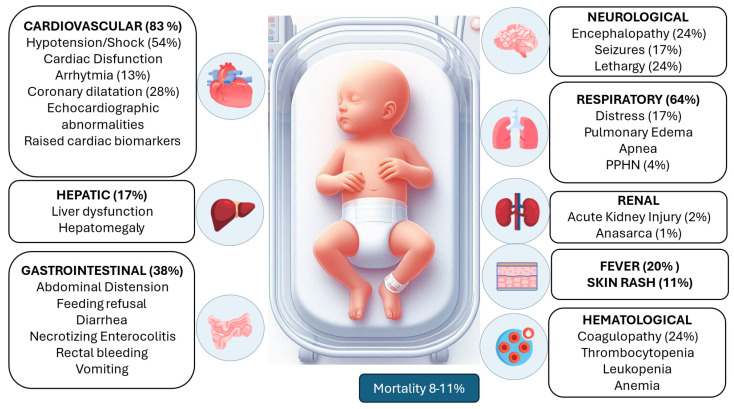
Multisystemic manifestation of MIS-N (modified from Mascarenhas et al. [63]).

MIS-N is characterized by a virus-induced antibody-mediated hyperinflammatory response that generally appears after 2–5 weeks from SARS-CoV-2 infection [59,60,61,62] with a wide spectrum of clinical manifestations, typically involving two or more systems (Figure 1).

The syndrome shares similarities with the multisystem inflammatory syndrome in children (MIS-C) but presents peculiar challenges due to unique physiological characteristics of neonates and the absence of a consensus definition or management for MIS-N. 

It was first described in 2021 [59], but since then, over 100 cases have been reported in the literature, most of them from India (about 90%) [63]. Its incidence is very rare amongst neonates with vertical transmission of COVID-19, but is 8% in symptomatic neonates [61]. The majority of the described infants were late preterm, with a slight prevalence in males [63].

The diagnosis is made by the exclusion of other conditions and is based on criteria adapted from the MIS-C [64], taking into account that fever rarely occurs in neonates and the source of primary infection is generally the mother [59,62,65]. These criteria are shown in Table 3.

Some authors [63,66] classified cases as “Most Likely”, “Possible”, and “Unlikely” based on the number and type of diagnostic criteria met. Moreover, cases of MIS-N can be divided in early (within the first 72 h) and late (after 72 h) based on the timing of presentation and, consequently, the potential source of SARS-CoV-2 antibodies [60,63,66].

Laboratory findings play a crucial role in the diagnosis of MIS-N [61]. These typically include elevated inflammatory markers, such as C-reactive protein (CRP), procalcitonin, erythrocyte sedimentation rate (ESR), IL-6, D-dimers, and ferritin. Cardiac biomarkers, including troponin and B-type natriuretic peptide (BNP), are important indicators of myocardial injury, while elevated liver enzymes and renal function tests may signal multiorgan involvement. The detection of SARS-CoV-2 antibodies, particularly IgG, in the absence of an active infection supports the diagnosis of MIS-N, indicating recent exposure to the virus.

Sometimes it could be hard to differentiate SARS-CoV-2 severe infection from MIS-N due to the simultaneous presence of antibodies and neonatal TNF positivity for SARS-CoV-2 [67]. Moreover, diagnosing MIS-N can be challenging due to the overlap with other neonatal conditions, such as bacterial sepsis, mycosis, viral infections, and birth asphyxia, which are more common in the neonatal period. Even maternal diseases should be considered, for example, maternal lupus.A differential diagnosis should include neonatal-onset multisystem inflammatory disease (NOMID, also known as chronic infantile neurologic cutaneous articular or CINCA syndrome) and neonatal Kawasaki disease, which both are very rare but similarly show widespread inflammation and multisystemic involvement [65].

The pathogenesis of MIS-N is not yet fully elucidated, but it is believed to be an exaggerated immune response triggered by SARS-CoV-2 infection. Three mechanisms have been proposed: exposure to maternal antibodies in utero; transplacental infection from maternal infection, leading to the endogenous production of antibodies in the fetus or child; and a post-infectious immune response to SARS-CoV-2 infection in a neonate [60,62,65,66]. Furthermore, in some genetically predisposed patients, circulating anti-SARS-CoV-2 antibodies and autoantibodies against various antigens, triggered by viral infection, may activate an inflammatory response cascade, leading to multi-organ involvement [68].

The role of maternal SARS-CoV-2 antibodies, transferred transplacentally, in modulating or exacerbating this response is a subject of ongoing research, with some studies suggesting that these antibodies might either protect against or contribute to the development of MIS-N [59,60,66].

Finally, all described cases were born to unvaccinated mothers [63]. In future, the dosage of an anti-spike Ig and anti-nuclear Ig could help to distinguish infection-induced MIS-N [62,66].

### 8.2. Neonatal Therapy

Symptomatic infants can benefit from supportive care, in most cases represented by oxygen and intravenous fluids, while specific anti-COVID-19 therapies and adjuvant therapies are not routinely recommended in the neonatal period. However, although remdesivir is only approved for children older than 28 days and who weigh more than 3000 g, case reports have been published of its successful use in younger and smaller patients on mechanical ventilation [69].

With regards to MIS-N, anti-inflammatory treatments, such as intravenous immunoglobulin (IVIg) and corticosteroids, often combined with supportive care may be beneficial, but at the moment, the emerging data are contradictory. In fact, some studies encourage routine use, while others suggest their limitation to severe cases due to the risk of infection and gastrointestinal issues [59,65]. Therefore, the treatment approach should be tailored based on the severity of the disease and the specific organ systems involved. In more severe cases, particularly those with significant cardiac involvement, biologic agents, such as anakinra, an IL-1 receptor antagonist, may be employed to control the hyperinflammatory state. Anti-platelet (aspirin) or anticoagulation therapy (unfractionated heparin or LMWH) may also be considered in neonates with significant coagulopathy or evidence of thromboembolic events [59,60,61,62,63].

Given the current knowledge about the potential negative effects on the newborn from a maternal SARS-CoV-2 infection, it is no longer considered necessary to prolong the hospital stay for a healthy newborn from a SARS-CoV-2-positive mother at the time of birth beyond the usual duration.

### 8.3. Hospital Stay

The separation of mother and infant has to be avoided and rooming-in should be encouraged if the neonatal and/or maternal clinical conditions (asymptomatic mother or with mild symptoms) permit it. The risk for the newborns of acquiring infection during the birth hospitalization from maternal infectious respiratory secretions can be reduced by precautions such as mask usage and other infection prevention measures, including washing hands and cleaning surfaces [4]. If noninfected partners or other family members are present during the birth hospitalization, they also should use face masks and practice hand hygiene when providing hands-on care to the infant.

Healthcare workers should use transmission-based precautions when caring for infants who are well in the same room as an infected mother and when caring for infants who are well at risk of SARS-CoV-2 infection.

Separation in a NICU is only necessary for neonates requiring intensive care. They should be optimally admitted to a single patient room with the potential for negative room pressure (or another air filtration system). If this is not available, or if multiple SARS-CoV-2-exposed infants must be cohorted, there should be at least 6 feet (2 m) between them, or they should be placed in air temperature-controlled isolettes that can provide an additional barrier against droplet transmission. Healthcare providers should use transmission-based precautions (a gown and gloves and use either an N95 respiratory mask and eye protection goggles or an air-purifying respirator that provides eye protection) for the care of infants requiring supplemental oxygen at a flow > 2 L per minute, continuous positive airway pressure, or mechanical ventilation [44].

The AAP identified several common scenarios with the following recommendations [44]:

▪ **Newborn infants who have been separated from an infected mother shortly after birth** and admitted directly to the NICU: infection control precautions should be used until a negative test is obtained within the first 72 h of age in order to determine the possible vertical transmission of the virus;
▪ **Newborn infants who have been rooming-in with an infected, presumed, or known contagious mother** who subsequently require admission to the NICU: infection control precautions should be used until 10 days have passed since the last maternal–infant contact, and testing on admission to the NICU and at 5 to 7 days after the last maternal contact are recommended in order to diagnose the possible horizontal transmission of the virus;
▪ **Healthy newborn infants born to positive mothers:** they should be tested at least once before hospital discharge, ideally close to the time of discharge in order to provide the most accurate family guidance;
▪ **Infants with ongoing care in the NICU who have a positive result on their initial testing**: consider follow-up testing at 48- to 72-h intervals until two consecutive negative tests are obtained to demonstrate that the virus has been cleared from mucosal sites;
▪ **Infants who require ongoing hospital care**: Caregivers should continue to use appropriate transmission-based precautions until discharge or until the infant has two consecutive negative test results collected ≥24 h apart. This approach should be reserved for sick and preterm newborn infants.

Three common scenarios may occur related to parents and caregivers of NICU infants, as summarized by the AAP [44]:

▪ **Pregnant persons who test positive for COVID-19 during routine obstetric testing but without symptoms or known exposure:** They should not be allowed to enter the NICU for 5 days after the positive test (entrance on day 6), as long as they remain asymptomatic. They should wear a face mask on days 6 through 10 after a positive test.
▪ **Mothers and partners who test positive for COVID-19 by PCR-based or antigen-based testing following symptoms or close exposure to an infected person:** They should not enter the NICU while able to transmit SARS-CoV-2. Immunocompetent people may be considered noninfectious if (a) afebrile for 24 h without the use of antipyretics, (b) at least 10 days have passed since the symptoms first appeared, and (c) symptoms have improved. People who are severely or critically ill should not enter the NICU until at least 20 days have passed since the symptoms first appeared or the first positive test.
▪ **Mothers and partners who are asymptomatic and were closely exposed to another infected person:** They can enter the NICU but wearing a face mask for 10 full days following the last close contact and were tested for SARS-CoV-2 at least 5 days following it. If they develop symptoms consistent with COVID-19 infection, they should be tested as soon as possible and not enter the NICU until their status is clarified.

Because recovered COVID-19 patients may have very prolonged (weeks to months) positive nucleic acid test results without evidence that such people remain infectious, centers should not require a negative PCR-based test before entry into the NICU.

### 8.4. Home Care

Newborn infants should be discharged based on each center’s routine criteria. According to the AAP [44]:

- **If the SARS-CoV-2 test result is positive** but the infant is asymptomatic, plans for frequent outpatient follow-up (either by phone, telemedicine, or in-office) through 14 days after birth should be established. During this period, precautions should be taken to prevent disease spread from infant to caregivers by using face masks and practicing hand hygiene in the home environment. Healthcare staff should use transmission-based precautions (or at least face masks and hand hygiene) in the outpatient office practice.
- **In most cases, the SARS-CoV-2 test result will be negative,** and infants will be discharged to families with exposed and maybe infected members. The mother should use a face mask and practice hand hygiene when directly caring for the infant, until she has been afebrile for 24 h without using antipyretics; at least 10 days have passed since the symptoms first appeared; the symptoms have improved and, in the case of an asymptomatic pregnant woman identified only by obstetric screening tests, at least 10 days have passed since the positive test. Other caregivers in the home should use face masks and practice hand hygiene before and after contact with the infant until their status is resolved.
- **If the infant cannot be tested**, then they should be treated as if SARS-CoV-2-positive for a 10-day period of observation. The mother should still maintain precautions until she meets the criteria for non-infectivity as described above.

Parents should be advised to pay close attention to the newborn’s health and contact the birth center and/or family pediatrician if symptoms such as fever, cough, rhinitis, drowsiness, vomiting, diarrhea, feeding difficulties, or respiratory issues appear. 

Once back home, the mother can initiate or continue breastfeeding and/or use expressed breast milk, depending on her general condition and desire, always wearing a mask. 

Although in-person post-discharge visits are preferable to provide timely newborn screening, bilirubin testing, and feeding and weight assessments, telemedicine and remote visits may be feasible after the early neonatal period [70]. 

Given the greater emotional vulnerability of pregnant and postpartum women, it is recommended to provide particular support to parenting, paying special attention to identifying situations of social distress and vulnerability and activating targeted interventions in agreement with local services

### 8.5. Outcomes

For infants with MIS-N, follow-up should be individualized, with specific attention to cardiology surveillance until more data are collected. Despite its severity, the prognosis for neonates with MIS-N is generally favorable with appropriate and prompt treatment. On the other hand, mortality in infants diagnosed with MIS-N is very high (range of 8–11%, depending on reports) [26,59,60,61,62,63]. 

Growth and developmental milestones should be followed closely in infants diagnosed with COVID-19 during the neonatal period because of emerging concerns regarding long-term neurodevelopmental outcomes. In fact, Goyal et al. demonstrated a mild motor delay (35%), mild language delay (40%), and moderate language delay (10%) in a small cohort of Indian newborns who acquired SARS-CoV-2 infection during the first wave of the pandemic (April–July 2020) [71]. Similarly, in Turkey, Ergon et al. highlighted lower psychomotor developmental index scores and a greater mildly delayed performance at 18–24 months, which were more noticeable with the Delta variant [72]. In contrast, studies from Kuwait showed no differences in cognitive outcomes at 18 months between infants diagnosed with COVID-19 in the neonatal period and those who had not been diagnosed [73].

## 9. Conclusions

In conclusion, both vertical and horizontal transmissions in the neonatal age appear to be less common and have a better prognosis than supposed at the beginning of the pandemic. Nevertheless, the hospital stay of a newborn from positive mother requires specific management protocols that can be difficult to apply in some centers, and the neonatal multisystemic inflammatory syndrome can be challenging to diagnose and manage. Whenever possible, breastfeeding has to be protected, and the separation of the dyad should be avoided. Moreover, appropriate protective equipment is recommended, both for infected women and for healthcare professionals. 

On the other hand, vaccination protects against adverse maternal–fetal outcomes and is now the most effective intervention for improving neonatal morbidity due to SARS-CoV-2 and can be administered at any time during pregnancy, with booster doses conferring additional protection, reassuring women in order to reduce vaccine hesitancy. More has to be performed to educate healthcare professionals and, subsequently, women on this topic.

## Figures and Tables

**Table 1:** Mother-to-child transmission (MTCT) of SARS-CoV-2 (modified by the WHO [24]).

	Maternal Infection	Fetal Exposure in Utero			Viral Persistence/ Immune Response	
	*Positive swab and/or symptoms*	PCR from sterile sample or not *	*Placental tissue*	*Serology* *(IgM or IgA)*	PCR from sterile sample or not *	*Serology* *(IgM or IgA)*
**In utero**	Anytime during pregnancy	Positive at age < 24 h	Positive at age < 24 h	Positive at age < 24 h	Positive at age 24–48 h	Positive at age 24 h to <7 days
***Intrapartum***	From 14 days prior to 2 days after birth	Negative at age < 24 h	Negative at age < 24 h	Negative at age < 24 h	Positive at age 24–48 h °	Positive at age 7–14 days §
**Early *post-natal***	From 14 days prior to 2 days after birth	Negative at age < 48 h	Negative at age < 24 h	Negative at age < 14 d	Positive at age ≥ 48 h §	Positive at age > 14 days §

* *sterile sample*: amniotic fluid, neonatal blood, lower respiratory tract samples obtained by bronchoscopic or non-bronchoscopic bronchoalveolar lavage, bronchial or tracheal aspirate, or cerebrospinal fluid; *non-sterile sample*: upper respiratory tract samples (e.g., nasopharyngeal or oropharyngeal swab or aspirate) or other non-sterile samples (e.g., stool). ° PCR from non-sterile sample confirmed by a positive PCR on a second non-sterile sample at age > 48 h to 7 day. § PCR from non-sterile sample confirmed by a positive PCR on a second non-sterile sample and serology confirmed on a second sample within 10 days.

**Table 2:** Summary of adjustments to the Neonatal Resuscitation Algorithm for neonates with suspected or confirmed SARS-CoV-2 infection (adapted from Edelson D et al., 2020 [46], and Morgan RW et al., 2022 [45]).

*Reduce provider risk* Effective use of personal protective equipment (PPE) for aerosol-generating procedures (AGPs). Receive the vaccine and recommended boosters against the SARS-CoV-2 virus.
*Provide timely, high-quality care while reducing provider exposure* Wear personal protective equipment (PPE) before entering the designated delivery/neonatal care room. Immediately excuse any initially unprotected resuscitation personnel and replace with providers wearing appropriate PPE for aerosol-generating procedures (AGPs). Perform endotracheal intubation, when indicated, after donning AGP-appropriate PPE. Minimize the number of providers to those actually needed to provide high-quality resuscitation. Communicate COVID-19 status of the patient/mother to any new providers and clearly communicate the expectations of wearing appropriate PPE.
*Specific strategies to reduce risk during CPR* Securely attach a HEPA filter to any ventilation device. Ventilate with a bag-mask and HEPA filter (as soon as available) with tight seal, ideally with a two-person technique, until an advanced airway is placed. Consider the use of a properly placed supraglottic airway to optimize chest compression fraction prior to intubation. Engage the physician airway provider with the highest chance of first-pass intubation success. Consider the use of video laryngoscopy if available and if personnel are appropriately trained. Maximize chest compression fraction, pausing to intubate only if needed. Minimize the endotracheal administration of medications to avoid aerosol generation. Minimize ventilation circuit disconnections.

**Table 3:** Currently used criteria for neonatal multisystemic inflammatory syndrome (MIS-N) (adapted from [59,63,66]).

1	Neonate aged <28 days at the time of presentation	
2	Evidence of SARS-CoV-2 infection in the mother	In the mother: Positive SARS-CoV-2 testing by RT-PCR, serology (IgG or IgM), or antigen during pregnancy. Symptoms consistent with SARS CoV-2 infection during pregnancy. COVID-19 exposure with confirmed SARS CoV-2 infection during pregnancy.
	OR	OR
	History of previously confirmed neonatal SARS-CoV-2 infection	In the neonate: Serological evidence of maternal infection (positive IgG specific to SARS CoV-2 but not IgM) in the neonate. Presence of SARS-CoV-2 antibodies (either IgG or IgM) in the neonate and a negative SARS-CoV-2 antigen test during the presentation to rule out an active COVID-19 infection.
3	Clinical criteria	Severe illness necessitating hospitalization AND Two or more organ systems affected [i.e., cardiac, renal, respiratory, hematologic, gastrointestinal, dermatologic, neurological, temperature instability (fever or hypothermia)] OR Cardiac AV conduction abnormalities OR coronary dilation or aneurysms (without involvement of a second organ system).
4	Laboratory evidence of inflammation	One or more of the following: Elevated CRP, ESR, fibrinogen, procalcitonin, D-dimer, ferritin, LDH, or IL-6. Elevated neutrophils or reduced lymphocytes. Low albumin.
5	No alternative diagnosis (such as birth asphyxia, viral or bacterial sepsis, and maternal lupus)	
